# Lung ultrasonography to diagnose community-acquired pneumonia in children

**DOI:** 10.1186/s12890-017-0561-9

**Published:** 2017-12-19

**Authors:** Nicola Principi, Andrea Esposito, Caterina Giannitto, Susanna Esposito

**Affiliations:** 1 Department of Pathophysiology and Transplantation, Università degli Studi di Milano, Milan, Italy; 2 Unit of Radiology, Fondazione IRCCS Ca’ Granda Ospedale Maggiore Policlinico, Milan, Italy; 3 Unit of Radiology, European Institute of Oncology, Milan, Italy; 4 Paediatric Clinic, Department of Surgical and Biomedical Sciences, Università degli Studi di Perugia, Piazza Menghini 1, 06129 Perugia, Italy

**Keywords:** Chest radiography, Community-acquired pneumonia, Lung ultrasonography, LUS, Ultrasound

## Abstract

**Background:**

Early diagnosis of community-acquired pneumonia (CAP) is essential to reduce the total burden of this disease. Traditionally, chest radiography (CR) is used to identify true CAP. However, CR is not a perfect diagnostic test for CAP. The use of lung ultrasonography (LUS) has been suggested as an alternative to overcome the problems associated with CR and increase the feasibility and accuracy of CAP diagnosis. LUS has largely been used for the diagnosis of several lung problems, including CAP, in adult patients with satisfactory results. Experience with LUS in children has grown over recent years. The main aim of this paper is to discuss the advantages and limits of LUS in the diagnosis of paediatric CAP.

**Discussion:**

The presence of a consolidation pattern during LUS may represent pneumonia or atelectasis, although this conclusion is operator dependent. An overall agreement between LUS and CR was observed in most of the studies that were examined. In most reports where a disagreement between the two methods was found, CR was not able to identify the cases that were correctly diagnosed by LUS, particularly when CR was performed only with postero-anterior/antero-posterior projection and consolidation was observed in lung areas that are poorly visualized by CR. However, the lack of standardized LUS methods is problematic. Finally, the real advantage of LUS for the diagnosis of CAP in children remains unclear.

**Summary:**

LUS is an interesting diagnostic modality that appears a useful first imaging test in children with suspected CAP. However, the methods used to perform LUS in children are not precisely standardized, and the diagnosis of interstitial CAP is inaccurate. Further studies are needed before LUS can be routinely used in everyday paediatric practice.

## Background

In recent decades, the incidence of community-acquired pneumonia (CAP) in the paediatric population has significantly decreased. It is likely that the widespread use of effective preventive measures, such as conjugate vaccines against *Haemophilus influenzae* type b and *Streptococcus pneumoniae*, two of the most common bacterial pathogens that cause paediatric CAP, has played a major role in this regard [1]. However, CAP remains a common cause of paediatric morbidity and mortality. Mortality due to CAP is significantly higher in the developing world, where approximately one million children younger than five years of age die annually due to this disease [2]. However, in developed countries, where only children with chronic severe underlying disease are at high risk of death, several million cases of CAP in otherwise healthy children are diagnosed yearly and cause significant medical, social and economic problems. Although most of these diseases are mild and can be treated at home, many cases result in hospitalization and, in some instances, the need for admission to the intensive care unit [3].

Early diagnosis of CAP is essential to reduce the total burden of the disease. In many cases, clinical signs and symptoms strongly suggest the diagnosis. However, in a significant number of children, CAP remains a diagnostic challenge mainly because a number of viral respiratory diseases, which require a different therapeutic approach, mimic the clinical manifestations of CAP [4]. Traditionally, chest radiography (CR) has been used to identify true CAP. However, CR is not a perfect diagnostic test for CAP. To explore all lung anatomy, at least two projections are needed. If only the postero-anterior/antero-posterior projection is used, some lung areas can be obscured by the heart, mediastinum and diaphragm [5]. The two projections approach leads to substantial exposure to ionizing radiation and a potential risk of cancer and gene mutation development, particularly in the youngest patients [6]. Moreover, image interpretation can vary significantly among observers, and delays in obtaining and processing images regularly occur [7]. Finally, CR results in poor image quality and is frequently unavailable in resource-poor settings.

All of these limitations explain why the use of CR for CAP diagnosis is not routinely suggested by experts and scientific societies [8–10]. CR is recommended only in selected cases, particularly those with severe clinical manifestations suggesting complications. Moreover, to reduce radiation exposure, only one projection, the postero-anterior/antero-posterior projection, is suggested by the majority of the experts, although this leads to an increased risk of missing CAP diagnoses [8, 10]. Finally, CR has to be avoided at the end of treatment to confirm a cure [8–10]. However, despite these statements, in clinical practice a number of CR significantly larger than that expected is usually performed [11, 12].

The use of lung ultrasonography (LUS) has been suggested to be able to overcome these limitations and increase the feasibility and accuracy of CAP diagnosis [13, 14]. LUS is fast, radiation-free, repeatable, inexpensive and easily performed at the bedside. In addition, LUS has been largely used for the diagnosis of several lung problems, including CAP, in adult patients with satisfactory results [15–17]. Experience with LUS in children has grown in recent years. The main aim of this paper is to discuss the advantages and limitations of LUS for the diagnosis of paediatric CAP.

## Discussion

### Use of ultrasound (US) to evaluate lung diseases

Point-of-care US has been used in adults by emergency physicians since the 1990s when the American College of Emergency Physicians published a position statement supporting the use of US by appropriately trained physicians [18]. Paediatric medicine later adopted this technology, and guidelines on the use of US by paediatricians were not published until recently [19]. However, the use of US in children has rapidly increased as evidenced by the results of a survey carried out in the USA in 2011, which reported that 95% of emergency departments with a paediatric emergency medicine program used this technology in some manner [20]. The evaluation of traumas and abdominal, renal, pelvic, scrotal, musculoskeletal and pancreaticobiliary diseases has been largely facilitated by the use of US. However, initially, lung diseases were not included among those conditions for which US could make a fundamental diagnostic contribution, whereas it has been now clarified that interpretation of LUS involves both directly observing lung structures (i.e., consolidations) and differentiating between normal artifacts (A lines) and abnormal artifacts that represent pathology (B lines). In healthy subjects, only the pleura is clearly visible as an echogenic line that moves accordingly with respiration. The so-called A-lines represent reverberations of the pleural line and are curvilinear, parallel lines that occur at regular intervals along the pleural line. Other vertically oriented lines arising from the pleural line and defined as B-lines can also be detected. Artefacts without clinical relevance include the vertical lines that do not reach the edge of the screen. A comparison of findings from lung computed tomography (CT) with those derived from transthoracic LUS established that B-lines are correlated with the thickening of the subpleural interlobular septa and expression of interstitial disease, including interstitial pneumonia [21, 22]. The number of B-lines tends to increase along with the decreasing air content of the lung. When consolidation occurs, a consolidative pattern is seen. The presence of a consolidation pattern, i.e., an hypoechogenic area with poorly defined borders and with vertical artefacts in the adjacent areas, may represent pneumonia or atelectasis. Although very difficult when the area of consolidation is small, differentiation remains possible via observation of the respiratory variance in air bronchograms, which are branching echogenic structures present in the consolidated area [23]. However, it should be highlighted that any CAP must be extended to the pleural surface to be diagnosed by LUS because examination of the central structures is prevented by the barrier created by the pleural–lung interface. Moreover, consolidation has to be within an intercostal window. This means that a small number of CAP cases will not be identified by LUS. Studies in adults seem to indicate that this can occur in approximately 8% of patients [24]. Moreover, it cannot be forgotten that LUS is operator dependent and poorly identifies the characteristics of the mediastinal and hilar opacities as well as those of the lung apex, lower left lobe and subscapular areas [25].

However, because a liquid allows for excellent propagation of sound waves, LUS is a very good modality to assess the presence of parapneumonic pleural effusions. LUS can detect physiologic amounts of pleural fluid [26], although a minimal volume of 20 mL in adults is more reliably detected [27], and all cases are correctly diagnosed when the liquid volume is greater than 100 mL [28]. LUS is significantly more sensitive than standard posterior-anterior CR, which in adults can detect blunting of the costophrenic recesses and obliteration of the hemidiaphragm only when >200 mL and >500 mL of fluid have accumulated, respectively [29]. Consequently, a few cases of effusion are missed, including some for which drainage is needed [30]. Moreover, it has been reported that LUS is more sensitive than a CT scan for the detection of minimal pleural effusion and septations within the pleural fluid [25]. Finally, although LUS cannot be substituted for pleural tapping in differentiating transudate from exudate, it can indicate the complexity of effusion according to the amount of floating particles, fibrinous strands, septations, loculations and fronds detected in the exudate [31]. The evidence of complex pleural effusion associated with a thickened pleura can be considered to be a strong indication for an immediate aggressive treatment [32].

Generally, in children, transthoracic LUS examination is performed using machines equipped with a high-resolution 5–15 MHz linear probe and 3–6 MHz convex probe. In neonates, particularly those that are small for their gestational age, slightly higher frequency probes, which are more effective in visualizing the chest wall, pleura, and lung peripheral parenchyma, can be used [33]. Most sonographers follow the scanning technique described by Copetti and Cattarossi [34]. Older infants, pre-school and school-age children are examined in the lateral decubitus and sitting position with the probe placed in the intercostal spaces perpendicular, oblique and parallel to the ribs in the anterior, lateral and posterior (lower and upper) thorax. In neonates, the supine, lateral and prone positions are used. The conventional B-mode US is routinely used. Colour Doppler sonography can provide data on vascular flow. The M-mode documents pleural or diaphragmatic motion.

In contrast to conventional radiology, LUS is a simple technique that is easy to learn. Physicians of different specialties can use LUS and correctly interpret the images even after a few hours of practice. Although some studies have reported that highly skilled physicians had a higher specificity in diagnosing CAP than less skilled physicians [33, 35–37], several reports have clearly demonstrated that a short training period can allow novice users to obtain satisfactory results. In the study by Esposito et al., a paediatric resident who underwent a one-day LUS training session was able to obtain sensitivity, specificity, and positive and negative predictive values for LUS of 97.9%, 94.5%, 94.0% and 98.1%, respectively, in comparison with CR [14]. Moreover, Bedetti et al., who compared LUS findings from a trained sonographer with ≥2 years of expertise in LUS assessment with those from a sonographer with training limited to 30 min, reported no substantial differences in the diagnostic abilities of the two physicians [38]. Finally, Zhan et al. showed that a paediatric resident with marginal LUS experience could identify CAP through evidence of consolidation with good specificity (91%; 95% confidence interval [CI] 83%–96%) and a positive likelihood ratio (4.71; 95% CI 2.21–10.04) [39]. In this study, the sensitivity was lower than that obtained with CR, but this was only partially ascribed to the poor experience of the sonologist. The main reasons of these poor results were considered the lack of any expert supervision and no knowledge of the patient’s signs and symptoms.

### Lung ultrasonography (LUS) for the diagnosis of community-acquired pneumonia (CAP) in children

In recent years, a number of studies have evaluated whether LUS can be considered to be a reliable alternative to CR for the diagnosis of CAP in children. In general, the conclusions were very favourable because the sensitivity, specificity, and positive and negative predictive values obtained using LUS were similar or sometimes better than those with CR [39–43]. An overall agreement between LUS and CR was demonstrated in most studies. Moreover, in most reports in which a disagreement between the two methods was found, CR was not able to identify the cases that were correctly diagnosed by LUS, particularly when CR was performed only with a postero-anterior/antero-posterior projection and consolidation was observed in lung areas that were poorly visualized by CR. Iorio et al. studied 29 hospitalized children aged 2 months to 12.5 years in whom the final diagnosis of CAP was based on CR, physical examination, and laboratory and instrumental tests as well and retrospectively compared the abilities of LUS and CR to diagnose CAP [35]. Agreement between the two methods was found in 24 cases. Four patients with negative CR had positive results by LUS, and only one child who was negative by LUS was positive by CR. False-negative CR cases identified on LUS were either located in the retrocardiac or diaphragmatic areas or were tested immediately after illness onset. Similar findings were previously reported by Copetti and Cantarossi who enrolled 79 children 6 months to 16 years in age with clinical signs suggestive of CAP and found LUS and CR positive results in 60 and 53 patients, respectively [34]. Disagreement was, at least in part, explained by the higher CAP detection rate of LUS because 4 of the children diagnosed by LUS were shown to be positive by CT. Iuri et al., studying 28 children 4 months to 17 years in age with clinical signs suggestive of CAP, found pleural effusion by LUS in 15 cases, but these results were confirmed by CR in only eight patients [35]. Caiulo et al. enrolled 102 children 1 to 16 years in age with clinically suspected CAP [36]. The disease was finally diagnosed by two independent, experienced paediatricians on the basis of clinical presentation in 89 patients. LUS and CR were positive for CAP in 88 and 81 children, respectively. One patient with normal LUS had positive CR results, whereas 8 patients with negative CR results had positive LUS. Pleural effusion was detected in 16 and three cases, respectively, by LUS and CR.

Moreover, LUS has been confirmed to be a valuable method for monitoring the evolution of lung consolidation and pleural effusion. Ho et al. [44] and Caiulo et al. [36] reported a progressive reduction in consolidations within a few days after diagnosis, and it has been supposed that monitoring the volume of LUS findings could be used to evaluate response to antibiotic administration [36].

However, accurate analysis of the data collected by these and other studies seems to indicate that not all of the problems regarding the routine use of LUS for CAP diagnosis in children have been solved and that, in some cases, less satisfactory results can be obtained via the use of LUS versus CR. Most of these problems are clearly evidenced in the meta-analysis carried out by Pereda et al. [45]. These authors analysed the papers that had acceptable methodologic quality (8 total) that they were able to select from the literature regarding CAP diagnosed by US in children published in 2014 or before. In the conclusions, the authors highlighted that the studies were very heterogeneous regarding the reference standard used to identify pneumonia, population enrolled and characteristics of the LUS technique. Moreover, the total numbers of global patients included in each of these analyses were relatively low. The pooled global population from these studies was limited to 765 children. The lack of a universal gold standard seems to be critical because the results of the comparison between LUS and CR are strongly influenced by the criteria used to confirm CAP cases. For example, in the meta-analysis, three studies used CR alone as a reference [13, 14, 35], whereas in the remaining 5 studies, CR was associated with blood results and/or clinical criteria [33, 34, 36, 37, 46]. The overall pooled sensitivity and specificity of LUS for the diagnosis of CAP were 96% (95% CI 94%–97%) and 93% (95% CI 90%–95.7%), respectively. However, when sensitivity and specificity were estimated only using CR, the sensitivity remained at 96% (95% CI 94%–98%), whereas the specificity was reduced to 84% (95% CI 80%–88%), demonstrating the poor ability of CR to be used in CAP diagnosis. On the other hand, it is well known that the gold standard method for evaluating the diagnostic value of an imaging technique of the lung is CT [47]. However, CT is not always feasible, cannot be performed at the point-of-care, is expensive and requires intensive exposure to radiation. Consequently, it cannot be routinely used for the evaluation of LUS and CR diagnostic properties. Only one study used CT in this manner. Ambroggio et al. prospectively studied children 3 months to 18 years of age who underwent CT for a clinical reason and in whom CR and LUS were also performed [48]. They performed CT in 27% (*n* = 36) patients, whereas the other subjects were placed into groups with ‘likely’ similar CT findings based on statistical analysis. On the entire cohort, these authors found that LUS and CR had similar sensitivities in detecting lung abnormalities, although LUS was statistically significantly more sensitive than CR in identifying normal lung anatomy (73% vs. 46%). By contrast, CR was statistically significantly more specific than LUS for the detection of both normal lung anatomy and lung pathologies. However, when only those patients who had a CT were analysed, many of those statistical differences were not seen. In that analysis, CR was more sensitive only in the detection of “other” lung pathologies, and more specific regarding only pleural effusion and interstitial disease.

The poor ability of LUS to predict interstitial CAP was associated with a very low interrater reliability and has been ascribed to the lack of agreement among sonographers on the number of B-lines and the distance among them needed to define interstitial disease [34, 49, 50]. However, this limitation is likely unimportant in clinical practice. Interstitial CAP is frequently a viral disease, has generally a good prognosis in otherwise healthy children, and does not require antibiotic treatment. More important might be the lower positive predictive value of LUS in comparison with CR for consolidation, a finding that is considered to be usually a marker of a bacterial CAP [51] and has been found to be almost systematically associated with a sustained increase in the neutrophil count and C-reactive protein concentration, as is common in bacterial infection [52]. However, the superiority of CR to LUS found in this study was obtained using both postero-anterior and lateral projections, a technique that increases the diagnostic ability of CR but is uncommon in clinical practice.

A second problem in most published studies is the inclusion of children with very different ages and body sizes. Two of the studies included in the meta-analyses enrolled only neonates, and in these subjects, LUS had a sensitivity of 96% (95% CI 90%–98.5%), which was not different from that calculated from whole pooled population, but a specificity of 100% (95% CI 92%–100%), which was significantly higher relative to the pooled population. The thoracic size of newborn infants is smaller than that of older children, which could lead to improved visualization of the lung parenchyma by LUS [53].

A third problem evidenced by the meta-analysis was the lack of standardized LUS methods. The approach described by Copetti and Cantarossi [34], which is probably the best method for obtaining optimal results from LUS, was used in only 5 of the 8 studies, and the procedure duration was reported only in 2 studies [14, 46].

Finally, as highlighted by Audette and Parent [47], the real advantage of LUS for the diagnosis of CAP in children remains unclear. Data regarding the reduction of CR use and the impact on patients are scant. Jones et al. conducted a randomized controlled trial in 191 patients from birth to 21 years of age with suspected CAP. The investigational group was subjected to LUS and CR only in cases of clinical uncertainty [54]. By contrast, the control group underwent both LUS and CR. The use of LUS led to a 38.8% reduction (95% CI 30%–48.9%) in CR in the investigational group with no cases of missed CAP. This resulted in an overall cost reduction of $9200. However, a slight but significant difference in antibiotic use was found in children who received LUS relative to those who underwent CR (37.9% vs. 27.3%; *p* > 0.05). This finding seems to be strictly related to the greater ability of LUS to detect very small consolidations that are not seen by CR and, consequently, to the greater number of CAP cases with a presumed bacterial aetiology diagnosed by LUS. In this study, consolidations ≤1 cm were diagnosed in 14.6% of the cases by LUS and in 4.5% by CR. It is not clear whether the differential detection of minor consolidations is an advantage because adequate therapy for presumed bacterial cases leads to greater antibiotic consumption. In this study, a minority of children with subcentimetre consolidations suffered from a very severe disease requiring hospitalization, oxygen administration and intravenous antibiotic therapy, suggesting that small consolidations can cause relevant clinical problems.

## Conclusions

LUS is an interesting diagnostic modality that offers several advantages in comparison with CR for the screening of CAP in children. Recent studies have been suggested that LUS could be used as the first imaging test in children with suspected CAP [55]. However, not all problems related to LUS use have been solved. The methods for performing LUS in children are not precisely standardized. Diagnosis of interstitial CAP by LUS is inaccurate, and the importance of small consolidations is not defined. Finally, the real impact in clinical practice of substituting CR with LUS has not been adequately studied. Further studies are needed before LUS can be routinely used in everyday paediatric practice.

## Abbreviations

CAP: community-acquired pneumonia
CI: confidence interval
CR: chest radiography
CT: computed tomography
LUS: lung ultrasonography
US: ultrasound.
